# Glycemic Variability and Diabetic Neuropathy in Young Adults With Type 1 Diabetes

**DOI:** 10.3389/fendo.2020.00644

**Published:** 2020-09-23

**Authors:** Marie Mathilde Bjerg Christensen, Eva Elisabeth Hommel, Marit Eika Jørgensen, Jesper Fleischer, Christian Stevns Hansen

**Affiliations:** ^1^Department of Clinical Epidemiology, Steno Diabetes Center Copenhagen, Gentofte, Denmark; ^2^Steno Diabetes Center Copenhagen, Gentofte, Denmark; ^3^Department of Population Health and Morbidity, Health in Greenland, University of Southern Denmark, Odense, Denmark; ^4^Institute of Nursing and Health Science, University of Greenland, Nuuk, Greenland; ^5^Steno Diabetes Center Aarhus, Aarhus, Denmark

**Keywords:** type 1 diabetes, glycemic variability, young adults, cardiovascular autonomic neuropathy, distal symmetric polyneuropathy, continuous glucose monitoring

## Abstract

**Background:** Glycemic variability (GV) may attribute to the pathogenesis of diabetic neuropathy. The aim of this cross-sectional study was to investigate the association between GV and distal symmetric polyneuropathy (DSPN) and cardiovascular autonomic neuropathy (CAN) in a Danish population of young adults with type 1 diabetes.

**Methods:** Young adults between 18 and 24 years with type 1 diabetes were included in this cross-sectional study. CAN was assessed by cardiovascular autonomic reflex tests (CARTs) and heart rate variability (HRV). DSPN was assessed by light pressure, pain and vibration perception, electrochemical skin conductance, sural nerve conduction velocity (SNCV), and amplitude potential (SNAP). GV were obtained by continuous glucose monitoring including coefficient of variation (CV), SD, continuous overall net glycemic action (CONGA), and mean amplitude of glucose excursions (MAGE).

**Results:** The study comprised 133 young adults (43.6% males), mean age of 22 years (SD 1.6). Unadjusted, higher CV was associated with a decreased risk of sural nerve conduction (*P* = 0.03), abnormal SNAP (*P* = 0.04) and incidents of definite CAN (*P* = 0.04). Likewise, higher CONGA was associated with increasing incidents of subclinical DSPN (*P* = 0.03), abnormal SNAP (*P* = 0.01), and SNCV (*P* = 0.02). However, both associations were not statistically significant in the fully adjusted model. Higher MAGE was associated with slightly increasing measures of HRV (*P* = 0.03) but only when fully adjusted. When correcting for multiple tests significance was lost. A significant association was found between HbA1c and measures of both DSPN (*P* < 0.02) and HRV (*P* < *0*.03) in fully adjusted models.

**Conclusions:** No significant associations between GV and diabetic neuropathy were found after adjusting for risk factors and multiple tests. This suggests that GV may not be a risk factor for diabetic neuropathy in young adults with type 1 diabetes. However, long-term effects of GV excursions may still play a role in the pathogenic mechanisms leading to neuropathy in later life.

## Introduction

Distal symmetric polyneuropathy (DSPN) and cardiovascular autonomic neuropathy (CAN) are severe and common complications of type 1 diabetes (1, 2). DSPN and CAN are usually rare problems during childhood but may be present in adolescents and young adults (3, 4). Improved glycemic control in patients with type 1 diabetes may prevent and revert early stages of DSPN and CAN and slow the progression toward overt neuropathy (5–9). Thus, detecting and preventing diabetic neuropathy at an early stage is essential.

The Diabetes Control and Complications Trial (DCCT) (10) demonstrated that intensive glycemic control in type 1 diabetes monitored by HbA1c reduced the onset and progression of diabetic neuropathy. Glycated hemoglobin (HbA1c) is an integrated assessment marker of glycaemia in diabetes treatment (11) and is associated with increased risk of diabetic complications like neuropathy. However, some limitations arise when using measures of HbA_1c_ to evaluate the role of glucose variability (GV): HbA_1c_ depicts an average of blood glucose over 3 months and does not reflect incidents of hypo- and hyperglycemia on a daily basis (12). Hence HbA_1c_ may be an insufficient tool to monitor and treat dysglycaemia. Diurnal GV may contribute to the risk of diabetic complications beyond HbA1c (13). The international consensus panel from the Advanced Technologies & Treatments for Diabetes Congress in February 2017 recommends using data from continuous glucose monitoring (CGM) and the coefficient of variation (CV) as the primary measure to assess GV.

Data on the association between GV and diabetic neuropathy is inconsistent, scarce, and has not previously been investigated in young adults with type 1 diabetes by applying CGM (14–19). Several studies point to glucose fluctuation as a risk factor for CAN in adults with type 1 diabetes (15–17). However, studies on the association between GV and peripheral neuropathy are conflicting (14, 15), possibly due to inconsistent use of various measures and definitions to assess GV and neuropathy in different studies. While large GV has been associated to oxidative stress, GV may attribute to the pathogenesis of diabetic neuropathy despite conflicting reports (13, 20). Studies on the association between GV and CAN and DSPN in type 2 diabetes are limited. However, they do demonstrate that variability of HbA1c in particular but also measures of GV assessed from CGM are associated with both CAN and DSPN (21–25).

The aim of this study was to investigate the association between modifiable glycaemic risk factors of neuropathy including GV and early and possibly reversible signs of DSPN and CAN early in the life of type 1 diabetes where preventive measures may have a substantial effect later in life. This was done in a Danish population of young adults with type 1 diabetes using the newest recommendations for assessing GV and both novel and established measures for DSPN and CAN.

## Materials and Methods

### Study Population

The study was designed as a cross-sectional observational study. The structure has been described in detail previously (4).

In order to investigate a population with early signs of diabetic neuropathy a cohort of young adults was assessed. All participants (age between 18 and 24 years) were recruited from the outpatient clinic at Steno Diabetes Center Copenhagen, Gentofte, Denmark. Participants were included regardless of duration of type 1 diabetes. Three hundred and fifty participants received a written invitation to participate and were subsequently contacted by phone. Written informed consent was obtained prior to examination. Ethical approval was obtained from The Danish Research Ethics Committee (project id.: H-15006967) (26).

Participants were excluded from the present study if they were not able to wear the CGM or failed to measure and log their capillary blood glucose during the 5-days of CGM-monitoring. Moreover, examination of CAN was not performed if they were treated with beta blockers.

There was no basis for conducting sensible power calculations to estimate a sample size for the aim of the study, because the investigated associations have not previously been investigated in young adults with type 1 diabetes with the use of CGM. Moreover, previous studies in other patient groups have demonstrated conflicting results.

### Assessment of Diabetic Neuropathy

DSPN was assessed and categorized according to recommendations made by the Toronto Diabetic Neuropathy Expert Group (2). Questionnaires were used to assess symptoms of DSPN (see section “Questionnaires on peripheral neuropathy and exposures” for further details).

Signs of DSPN were evaluated by established measures: Light pressure perception was assessed by applying a 10-g monofilament until it buckled (Neuropen®, Owen Mumford Ltd, Oxford, UK) to three points at the distal bilateral foot pads: just proximal to the great, third and fifth toe (27, 28). Pain sensation was evaluated by using a 40-gram pin prick device (Neuropen®, Owen Mumford Ltd, Oxford, UK) applied at the dorsal side of the toes just proximal to the nail on the great, third, and fifth toe. Vibration perception threshold (VPT) was determined using a Bio-Thesiometer (Bio-medical instruments, Ohio, USA) at the distal end of the great toe on both feet, and age stratified perception thresholds were used to assess abnormal results (29).

Novel measures were used to objectively evaluate DSPN: Small autonomic fiber function was assessed using the non-invasive device Sudoscan™ (Impeto Medical, Paris, France) performing an electrochemical skin conductance (ESC) test on the hands and feet (30). Age and gender stratified ESC thresholds were applied (31). Sural nerve conduction velocity (SNCV) and amplitude potential (SNAP) were obtained by the handheld NC-Stat® DPNCheck^TM^ (NeuroMetrix, Inc., Waltham, USA) (32). Age and height stratified SNAP and SNCV thresholds were used (33). Participants were examined for bilateral abnormalities in SNAP and SNCV. A composite measure, “sural nerve conduction” (SNC), was used when abnormalities in either SNAP, SNCV or both bilaterally. DSPN was defined according to four categories. The label “possible DSPN” was given if presence of symptoms of peripheral neuropathy as assessed by questionnaires, or signs as assessed by VPT, light pressure and pain perception were confirmed. If presence of symptoms and signs were confirmed the label “probable DSPN” was added. “Confirmed DSPN” was given if either the test for SNC or ESC was abnormal and if the participants had symptoms or signs. Ultimately, “subclinical DSPN” was defined as presence of abnormal SNC or ESC without symptoms or signs.

To evaluate CAN three standard cardiovascular autonomic reflex tests (CARTs) and measures of 5 min. resting heart rate variability (HRV) were performed in a quiet examination room. HRV was assessed after 5 min of supine rest and analyzed from 5-min resting heart rate (HR). HRV indices were analyzed in time- and frequency-domain. Time-domain included the root mean square of the sum of the squares of differences between consecutive R–R intervals (RMSSD) and standard deviation of normal-to-normal intervals (SDNN). Frequency-domain included low-frequency power band (LF) (0.04–0.15 Hz), high-frequency power band (HF) (0.15–0.4 Hz), total frequency power (Total) and the ratio low-frequency power/high-frequency power (LH/HF-ratio) (34).

The 5-min resting HRV test was followed by the three CARTs including the lying-to-standing test (30:15), the deep breathing test (E:I) and Valsalva Maneuver. “Early CAN” was defined as one out of the three CARTs was abnormal, “definite CAN” was defined as two or three were abnormal. Thresholds for abnormal results were age stratified (35). Resting HRV indices and CARTs were registered by using Vagus™ (Medicus Engineering, Aarhus, Denmark).

In line with the recommended criteria for examination of CAN (26), participants were asked to restrain from vigorous exercise 24 h before examination and from caffeine consumption on the specific day of examination.

### Questionnaires on Peripheral Neuropathy and Exposures

Each patient was asked to fill in the questionnaires Brief Pain Inventory (BPI) and Michigan Neuropathy Screening Instrument (MNSI) on the examination day. Participants were diagnosed with painful diabetic neuropathy if they in the BPI questionnaire answered having pain in both legs and/or both arms peripherally (36). A MNSI score of ≥7 was interpreted as presence of neuropathy (37).

Moreover, a questionnaire considering life style factors such as smoking status (current, former, or never) and weekly amount of exercise in hours (pooled light and moderate/vigorous exercise) was filled in.

### Assessment of GV Indices

The CGM sensor Enlite™ (Medtronic, Northridge, CA) was inserted into the subcutaneous tissue of the abdomen or alternatively the upper arm. Subsequently the iPro2™ (Medtronic, Northridge, CA) recorder was attached. The sensors should be worn for 5 days and the capillary finger blood glucose monitored four times daily for calibration. The software Medtronic CareLink™ iPro™ was used to generate data from the sensors. Participants were excluded from the study if there were not enough measurements of the capillary blood glucose to run the Medtronic CareLink™ iPro™ software. CV, standard deviation (SD), continuous overall net glycemic action (CONGA), and mean amplitude of glucose excursions (MAGE) were used to quantify GV (38). Time spent in hypo- (<3.0 mmol/l), eu- (≥3.0; ≤10.0 mmol/l), and hyperglycemia (>10.0 mmol/l) were calculated (38) and presented in minutes and percentage.

### Blood Pressure and Anthropometric Measures

Blood pressure and heart rate (HR) were measured after 10 min of rest and calculated as the mean of three consecutive measures performed with intervals of 1 min. Automated oscillometric blood pressure recorders were used (AND UA-787plus, A&D medical, California, USA).

Height and weight were measured with clothes on but without shoes using a fixed rigid stadiometer (Seca, Chino, USA) and an electronic scale (Mettler Toledo, Glostrup, Denmark), respectively.

### Biochemical Measures

All biochemical measures were analyzed from venous blood samples except for urine albumin and creatinine. Blood and urine samples were collected on the same day as the examination. The participants were non-fasting.

HbA_1c_ was analyzed by high performance liquid chromatography on a Tosoh G7 (Tosoh Cooperation, Japan). C-peptide was measured using a Cobas e411 (Roche Diagnostics, Mannheim, Germany). Triglycerides, HDL, and total cholesterol were analyzed by standard enzymatic colorimetry techniques on a Vitros 5600 (Ortho Clinical Diagnostics, France). Serum LDL cholesterol was calculated using the Friedewald equationTriglyceride level did not exceed 4.5 mmol/l in any subject. Hence, no other LDL assessments were deemed relevant. Plasma creatinine was analyzed by two-point rate enzymatic technique. The Chronic Kidney Disease Epidemiology (CKD-EPI) equation was used to estimate eGFR (39). Urinary albumin-to-creatinine ratio was analyzed by quantitative immunological turbidimetry.

### Medication

Data on medication were extracted from hospital electronic records and validated by the patient at examination day.

### Statistical Analysis

Patient characteristics are presented as means with standard deviation (SD) or in case of skewed distributions as medians with interquartile range [IQR].

Participants were excluded from the analysis of a specific test if the values were missing.

Both GV and HbA_1c_ were examined as determinants for neuropathy. The associations were assessed by logistic regression for the categorical outcomes and presented as odds ratios (OR) with 95% confidence interval (CI). Linear regression analyses were applied for continuous outcomes and presented as estimates with 95% CI. To meet model assumptions outcomes were log-transformed prior to analysis and subsequently back transformed to original scale where appropriate. To avoid small-sample bias, determinants of DSPN and CAN were not included in the analyses if the number of affected participants were <5.

Four models of adjustments were applied: Model 1: Unadjusted; Model 2: Adjusted for age and gender; Model 3: Adjusted as model 2 + diabetes duration, BMI, exercise and HbA_1c_; Model 4: Adjusted as model 3 + systolic blood pressure, triglycerides, LDL cholesterol and current smoking. HR was included as a confounder in models where HRV indices were determinants.

All analyses used 2-sided *P* = 0.05 as statistically significant and were adjusted for multiple tests by the Benjamini-Hochberg procedure (40).

Statistical analyses were performed in R version 3.3.3 (The R Foundation for Statistical Computing) and SAS, version 9.4 (SAS Institute, Cary, NC, USA).

## Results

### Patient Characteristics

Overall, 133 young adults (43.6% male) were included in the study. Twenty-three participants were excluded due to missing or lacking CGM-monitoring including insufficient numbers of capillary finger blood glucose monitoring. Reasons for not wearing a CGM sensor were primarily irritative/allergic reactions to the bandage patches or fear of discomfort. Mean (SD) age was 22 years (1.6), diabetes duration 11 years (5.2), and median (IQR) HbA_1c_ 65.5 mmol/mol (57;74). Mean (SD) BMI was 24.7 kg/m^2^ (3.8) and 122 (92.4%) participants exercised regularly for an average of 9 h weekly. All participants were treated with insulin. Participant characteristics are presented in Table 1.

**Table 1:** Characteristics of the study population.

**Clinical characteristics**	**Mean (*SD*)/Median [IQR]/*N* (%)**
***N***	**133**
Age (yr)	22 (1.6)
Males (%)	58 (43.6)
CSII treatment (%)	67 (50.4)
Diabetes duration (yr)	11.0 (5.2)
BMI (kg/m^2^)	24.7 (3.8)
Exercise (%) / (hr/week)	92.4 / 9.0 [5.0;15.5]
Current smoker (%)	28 (21.2)
Systolic blood pressure (mmHg)	125.9 (11.4)
Diastolic blood pressure (mmHg)	81.2 (8.6)
Heart rate (bpm)	76.6 (14.2)
**Biochemistry**	
HbA_1c_ (mmol/mol)	65.5 [57.0;74.0]
HbA_1c_ (%)	8.2 [7.4;9.0]
Cholesterol (mmol/l)	4.4 (1.1)
Triglycerides (mmol/l)	1.1 [0.8;1.6]
HDL (mmol/l)	1.3 (0.4)
LDL (mmol/l)	2.5 (0.9)
Urine albumin/creatinine ratio (mg/g)	6.0 [4.0;11.0]
eGFR (ml/min/1.73m2)	123.0 [115.9;127.1]
C-peptide (pmol/l)	14.5 [7.0;101]
**Medication**	
Insulin treatment *n* (%)	133 (100)
Metformin *n* (%)	1 (0.8)
Other glucose-lowering drugs *n* (%)	1 (0.8)
Antihypertensive treatment *n* (%)	6 (4.5)
Beta blocker treatment *n* (%)	2 (1.5)
Lipid lowering treatment *n* (%)	1 (0.8)
Psychotropics *n* (%)	5 (3.8)

*Data are given in means (SD), medians [IQR] or proportions %*.

*eGFR, Estimated glomerular filtration rate; BMI, Body Mass Index; HDL, High-density lipoproteins; LDL, Low-density lipoproteins*.

### Diabetic Neuropathy

The results of the prevalence of DSPN and CAN have been discussed elsewhere (4). In total, 51.1% (*n* = 68) were diagnosed with subclinical DSPN. One patient (0.8%) had confirmed DSPN, and two (1.5%) possible DSPN. None met the criteria for probable DSPN. Prevalence estimates of symmetric abnormal SNAP and SNCV were 20.3% (*n* = 26) and 34.4% (*n* = 44), respectively. Prevalence of the composite measure of SNAP/SNCV, SNC was 48.4% (*n* = 62). Abnormal ESC results on feet were found in 4.5% (*n* = 6) and 3% (*n* = 4) on hands. Symmetrically abnormal VPT was detected in 0.8% (*n* = 1) and likewise for symmetrical neuropathy diagnosed by the BPI questionnaire. No participants had abnormal results when light touch, pain perception or MNSI questionnaire were used.

Definite CAN was diagnosed in 6.1% (*n* = 8) and early CAN in 26.9% (*n* = 35).

Distribution and prevalence estimate of the outcomes are presented in Table 2.

**Table 2:** Distribution of outcome and GV measures and prevalences of abnormal results.

***N***		**133**
**GV measures**	**Mean (*SD*)/median [IQR]**	**Prevalence *n* (%)**
CV (%)	40 (10)	NA
SD (mmol/l)	3.9 [3.2;4.7]	NA
MAGE (mmol/l)	7.7 [5.9;9.9]	NA
CONGA (mmol/l)	9.1 (2.2)	NA
Time spent in hypoglycaemia (min.) / (%)	35 [0;120] / 1.0 [0.0;4.0]	NA
Time spent in euglycaemia (min.) / (%)	3065 [2125;3895] / 52.2 (19.6)	NA
Time spent in hyperglycaemia (min.) / (%)	2650 [1740;3480] / 44.7 (20.6)	NA
**Outcome Measures**		
**CAN Measures**		
CAN	NA	8 (6.1)
Early CAN	NA	35 (26.9)
Lying to standing ratio (30:15)	1.4 (0.2)	21 (15.9)
Deep breathing ratio (E:I)	1.5 (0.2)	10 (7.6)
Valsalva Maneuver ratio (VM)	1.7 (0.4)	22 (16.9)
SDNN (ms)	48.1 [36.3;68.2]	NA
RMSSD (ms)	38.9 [25.7;59.3]	NA
LF (ms^2^)	290.1 [130.1;670.0]	NA
HF (ms^2^)	251.3 [114.6;516.0]	NA
LF/HF ratio	1.3 [0.8;2.8]	NA
Total	779.5 [444.4;1570.5]	NA
**DSPN Measures**		
Subclinical DSPN	NA	68 (51.1)
Confirmed DSPN	NA	1 (0.8)
Possible DSPN	NA	2 (1.5)
Probable DSPN	NA	0
Monofilament (≥ 1 missing response)	NA	0
Pin prick (≥ 1 missing response)	NA	0
SNC	NA	62 (48.4)
SNAP (μV)	11.7 [8.7;15.0]	26 (20.3)
SNCV (m/s)	50.8 (4.2)	44 (34.4)
VPT (V)	4.5 [3.5;5.5]	1 (0.8)
ESC—hands (μS)	77.5 [69.5;83.5]	4 (3.0)
ESC—feet (μS)	82.3 [78.6;85.8]	6 (4.5)
**Questionnaires**		
BPI questionnaire:		
*Painful neuropathy (% answered yes)*	NA	1 (0.8)
MNSI questionnaire:		
*MNSI neuropathy score (score* ≥ *7 points)*	1 [0;2]	0

*Data are given in means (SD), medians [IQR], or proportions. NA, Not applicable*.

*CAN, Cardiovascular autonomic neuropathy; RMSSD, root mean square of the sum of the squares of differences between consecutive R–R intervals; SDNN, standard deviation of normal-to-normal intervals; LF/HF ratio, low-frequency power/high-frequency power ratio; DSPN, distal symmetric polyneuropathy; VPT, vibration perception threshold; SNC, sural nerve conduction; SNAP, sural nerve amplitude potential; SNCV, sural nerve conduction velocity; ESC, Electrochemical skin conduction; BPI, Brief Pain Inventory; MNSI, Michigan Neuropathy Screening Instrument questionnaire; CV, coefficient of variation; MAGE, mean amplitude of glucose excursions; CONGA, continuous overall net glycemic action*.

*Capillary blood glucose was divided into three groups: Hypoglycemia: <3.0 mmol/l, euglycemia: ≥3.0; ≤10.0 mmol/l, hyperglycemia: >10.0 mmol/l*.

### Glucose Variability

Mean (SD) CV was 40% (10) and median (IQR) SD was 3.9 mmol/l (3.2;4.7). Distribution of GV measures are presented in Table 2.

### Association Between Glucose Variability and Diabetic Neuropathy

#### Coefficient of Variation (CV)

**Figure 1:**
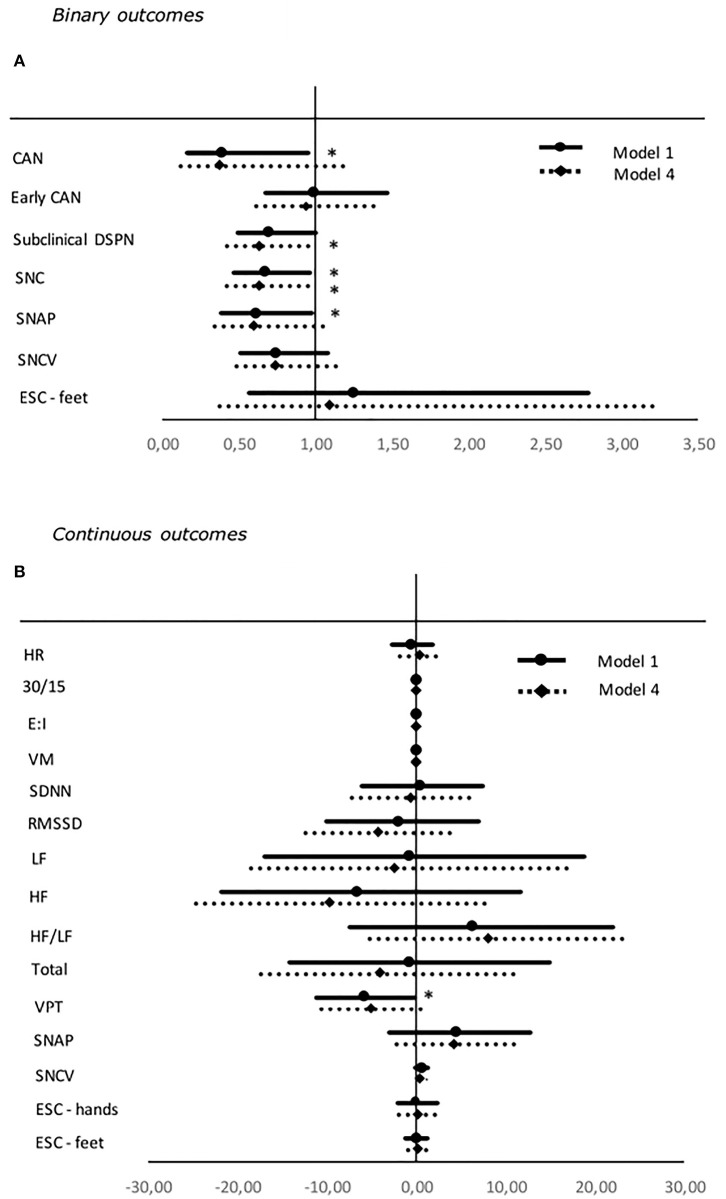
Forest plot of the associations between standardized values of CV and both binary **(A)** and continuous **(B)** neuropathy endpoints. For binary outcomes results are presented as odds ratio and 95% confidence intervals. Odds ratio shows the change in odds for an increase of one deviation in the HbA_1c_. For continuous outcomes results are presented as estimates and 95% confidence intervals. Estimates show the percentage change in the outcomes for an increase of one standard deviation in the SD. Studies with confidence interval crossing the vertical line are inconclusive. Model 1: unadjusted. Model 4: adjusted for age, gender, diabetes duration, BMI, exercise, systolic blood pressure, triglycerides, LDL cholesterol, and current smoking. SDNN, RMSSD, LF, HF, LF/HF ratio, and total are adjusted for HR in every model. Outcomes of DSPN are define as presence of symmetric abnormal results. Binary outcomes were only included in the analyses if presence of five or more abnormal events. CV, coefficient of variation; CAN, Cardiovascular autonomic neuropathy; SNC, sural nerve conduction; SNAP, sural nerve amplitude potential; SNCV, sural nerve conduction velocity; ESC, Electrochemical skin conduction; RMSSD, root mean square of the sum of the squares of differences between consecutive R–R intervals; SDNN, standard deviation of normal-to-normal intervals; LF/HF ratio, low-frequency power/high-frequency power ratio; VPT, vibration perception threshold. *P < 0.05.

Greater CV was associated with a decrease in incidents of symmetric abnormalities in SNAP, SNC, and definite CAN unadjusted and when adjusted for age and gender in model 2. Only for SNC significance remained in fully adjusted models. In addition, higher CV was inversely associated to incidents of subclinical DSPN in fully adjusted models (Table 3, Figure 1). However, both associations lost significance after applying the Benjamini-Hochberg procedure (40) to account for multiple testing.

**Table 3:** The association between CV and measures of diabetic neuropathy.

**CAN Measures**	**Model 1**	**Model 2**	**Model 3**	**Model 4**
**Binary outcomes**	**OR (95% CI)**			
CAN	0.0 (0.0;0.61)^*^	0.0 (0.0;0.56)^*^	0.0 (0.0;8.34)	0.0 (0.0;6.18)
Early CAN	0.96 (0.02;55.92)	0.97 (0.02;57.80)	0.87 (0.01;62.08)	0.49 (0.01;38.15)
**Continuous outcomes**	**Estimate (95% CI)**			
Heart rate	−4.46 (−28.32;19.41)	−4.11 (−27.60;19.38)	0.33 (−23.85;24.50)	4.77 (−19.06;28.61)
Lying to standing (30:15)	0.08 (−0.32;0.49)	0.09 (−0.32;0.49)	0.02 (−0.39;0.43)	−0.02 (−0.43;0.39)
Deep breathing (E:I)	0.08 (−0.34;0.50)	0.08 (−0.33;0.50)	−0.02 (−0.45;0.40)	0.01 (−0.40;0.42)
Valsalva Maneuver (VM)	0.07 (−0.57;0.71)	0.06 (−0.57;0.70)	0.15 (−0.51;0.80)	0.13 (−0.54;0.79)
SDNN	4.90 (−48.10;112.01)	5.97 (−47.52;113.97)	−1.16 (−51.48;101.37)	−6.04 (−54.08;92.26)
RMSSD	−18.11 (−66.90;102.59)	−18.30 (−66.66;101.56)	−25.56 (−70.56;88.17)	−36.08 (−74.82;62.22)
LF	−6.83 (−85.63;505.08)	−4.61 (−85.25;516.84)	−11.44 (−86.55;483.07)	−21.28 (−88.13;422.14)
HF	−50.65 (−92.32;217.14)	−49.85 (−92.03;215.73)	−57.18 (−93.48;181.17)	−65.48 (−94.77;127.68)
LF/HF ratio	88.76 (−55.36;698.18)	90.20 (−53.19;672.85)	106.85 (−48.65;733.21)	128.09 (−42.70;808.01)
Total	−6.86 (−79.70;327.46)	−5.53 (−79.42;333.52)	−26.26 (−84.37;247.82)	−34.85 (−86.34;210.86)
**DSPN Measures**				
**Binary outcomes**	**OR (95% CI)**			
Subclinical DSPN	0.03 (0.0;1.03)	0.02 (0.0;1.02)	0.01 (0.0;0.48)^*^	0.01 (0.0;0.78)^*^
SNC	0.02 (0.0;0.69)^*^	0.01 (0.0;0.58)^*^	0.01 (0.0;0.48)^*^	0.01 (0.0;0.81)^*^
SNAP	0.01 (0.0;0.80)^*^	0.0 (0.0;0.72)^*^	0.01 (0.0;1.83)	0.01 (0.0;1.97)
SNCV	0.05 (0.0;2.29)	0.04 (0.0;2.24)	0.03 (0.0;2.73)	0.05 (0.0;4.39)
ESC—feet	10.75 (0.0;43428.88)	14.86 (0.0;98876.15)	1.67 (0.0;52896.65)	2.61 (0.0;189015.80)
**Continuous outcomes**	**Estimate (95% CI)**			
VPT	−46.50 (−70.95;−1.48)^*^	−46.57 (−70.98;−1.62)^*^	−44.16 (−70.63;6.14)	−41.24 (−68.97;11.28)
SNAP	59.59 (−27.44;251.0)	60.87 (−20.46;225.35)	49.27 (−23.81;192.44)	54.88 (−20.55;201.92)
SNCV	6.33 (−1.15;13.82)	6.29 (−0.99;13.56)	6.47 (−0.47;13.40)	5.71 (−1.23;12.66)
ESC—hands	1.38 (−19.65;27.92)	1.13 (−19.63;27.27)	3.97 (−18.15;32.07)	3.72 (−18.46;31.94)
ESC—feet	0.13 (−12.07;14.01)	0.06 (−12.11;13.92)	2.96 (−9.98;17.77)	3.91 (−9.05;18.71)

*Results are presented as odds ratios for binary outcomes based on logistic regression analyses and estimates for continuous outcomes based on linear regression analyses. Odds ratios show the change in odds for any increase of the GV determinants. Estimates show the percentage change in the outcomes for every 1-unit change of CV*.

*Model 1: unadjusted. Model 2: adjusted for age and gender. Model 3: adjusted for age, gender, HbA1c, diabetes duration, BMI and exercise. Model 4: adjusted for age, gender, HbA1c, diabetes duration, BMI, exercise, systolic blood pressure, triglycerides, LDL cholesterol and current smoking. The continuous outcomes of SDNN, RMSSD, LF, HF, LF/HF ratio, Total, VPT, SNAP, and ESC for hands and feet are log-transformed prior to analysis and subsequently back transformed to original scale. SDNN, RMSSD, LF, HF, LF/HF ratio, and Total are adjusted for HR in every model. Outcomes of DSPN are defined as presence of symmetric abnormal results. Binary outcomes were only included in the analyses if presence of 5 or more abnormal events*.

*CAN, Cardiovascular autonomic neuropathy; RMSSD, root mean square of the sum of the squares of differences between consecutive R-R intervals, SDNN, standard deviation of normal-to-normal intervals; LF/HF ratio, low-frequency power/high-frequency power ratio, DSPN, distal symmetric polyneuropathy; VPT, vibration perception threshold; SNC, sural nerve conduction; SNAP, sural nerve amplitude potential; SNCV, sural nerve conduction velocity; ESC, Electrochemical skin conduction*.

* *P < 0.05*.

#### Standard Deviation (SD)

**Figure 2:**
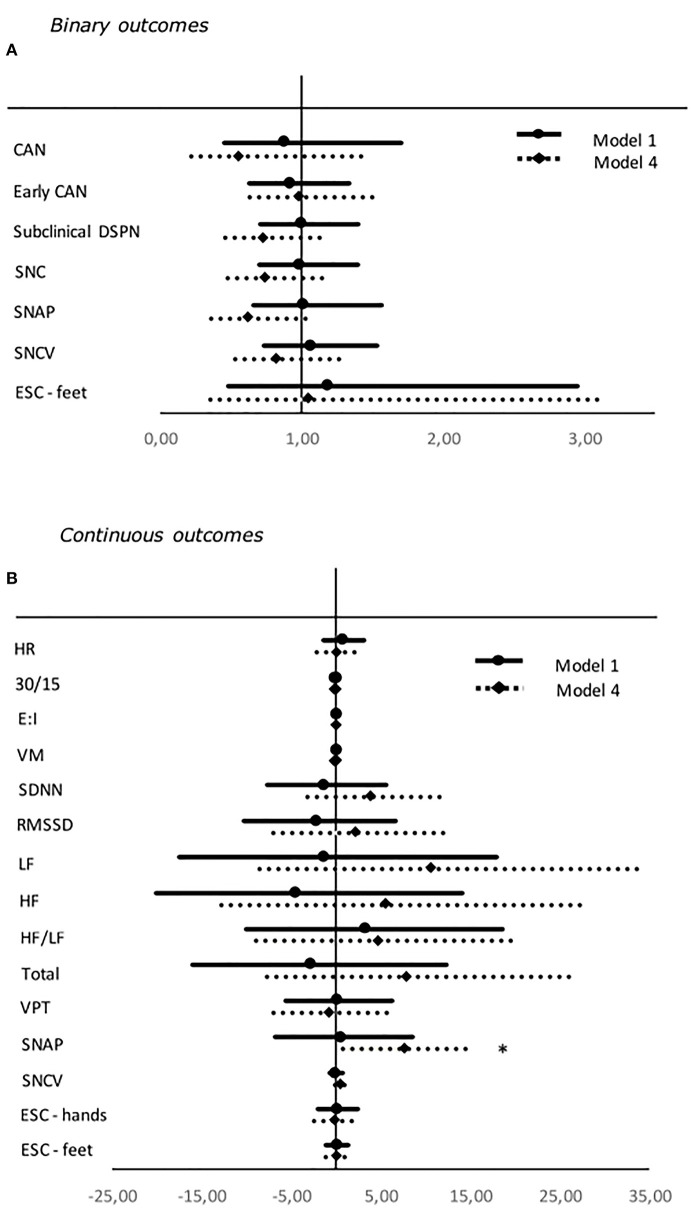
Forest plot of the associations between standardized values of SD and both binary **(A)** and continuous **(B)** neuropathy endpoints. For binary outcomes results are presented as odds ratio and 95% confidence intervals. Odds ratio shows the change in odds for an increase of one deviation in the HbA_1c_. For continuous outcomes results are presented as estimates and 95% confidence intervals. Estimates show the percentage change in the outcomes for an increase of one standard deviation in the SD. Studies with confidence interval crossing the vertical line are inconclusive. Model 1: unadjusted. Model 4: adjusted for age, gender, diabetes duration, BMI, exercise, systolic blood pressure, triglycerides, LDL cholesterol, and current smoking. SDNN, RMSSD, LF, HF, LF/HF ratio, and total are adjusted for HR in every model. Outcomes of DSPN are define as presence of symmetric abnormal results. Binary outcomes were only included in the analyses if presence of five or more abnormal events. CAN, Cardiovascular autonomic neuropathy; SNC, sural nerve conduction; SNAP, sural nerve amplitude potential; SNCV, sural nerve conduction velocity; ESC, Electrochemical skin conduction; RMSSD, root mean square of the sum of the squares of differences between consecutive R–R intervals; SDNN, standard deviation of normal-to-normal intervals; LF/HF ratio, low-frequency power/high-frequency power ratio; VPT, vibration perception threshold. *P < 0.05.

Higher SD was associated to a decreased risk of subclinical DSPN and increasing levels of the continuous outcome SNCV when adjusted for diabetes duration, BMI, and exercise and HbA_1c_ in model 3. Again, no significance persisted after correcting for multiple tests (Table 4, Figure 2).

**Table 4:** The association between *SD* and measures of diabetic neuropathy.

**CAN measures**	**Model 1**	**Model 2**	**Model 3**	**Model 4**
**Binary outcomes**	**OR (95% CI)**			
CAN	0.79 (0.41;1.51)	0.79 (0.41;1.53)	0.54 (0.23;1.28)	0.47 (0.17;1.32)
Early CAN	1.02 (0.71;1.45)	1.03 (0.72;1.48)	1.14 (0.76;1.71)	1.13 (0.75;1.72)
**Continuous outcomes**	**Estimate (95% CI)**			
Heart rate	0.67 (−1.41;2.76)	0.69 (−1.38;2.76)	−0.19 (−2.44;2.06)	0.29 (−1.93;2.25)
Lying to standing (30:15)	−0.02 (−0.06;0.01)	−0.02 (−0.06;0.01)	−0.01 (−0.05;0.02)	−0.02 (−0.06;0.02)
Deep breathing (E:I)	0.01 (−0.03;0.04)	0.01 (−0.03;0.04)	0.01 (−0.03;0.05)	0.01 (−0.03;0.05)
Valsalva Maneuver (VM)	0.03 (−0.02;0.09)	0.03 (−0.03;0.08)	0.02 (−0.04;0.08)	0.02 (−0.04;0.08)
SDNN	−2.87 (−8.66;3.29)	−2.65 (−8.48;3.55)	1.18 (−5.30;8.10)	1.41 (−5.12;8.39)
RMSSD	−4.10 (−11.39;3.80)	−4.11 (−11.40;3.77)	−0.26 (−8.50;8.71)	−1.06 (−9.28;7.90)
LF	−5.42 (−19.70;11.38)	−4.87 (−19.29;12.11)	2.77 (−13.74;22.46)	3.97 (−12.80;23.97)
HF	−9.43 (−23.02;6.55)	−9.29 (−22.83;6.63)	−1.53 (−17.39;17.36)	−2.47 (−18.23;16.33)
LF/HF ratio	4.43 (−7.98;18.52)	4.87 (−7.33;18.68)	4.38 (−8.33;18.86)	6.61 (−6.27;21.26)
Total	−6.60 (−18.23;6.69)	−6.35 (−18.08;7.07)	1.03 (−12.52;16.68)	1.56 (−12.16;17.43)
**DSPN Measures**				
**Binary outcomes**	**OR (95% CI)**			
Subclinical DSPN	0.94 (0.69;1.28)	0.93 (0.67;1.28)	0.67 (0.45;0.99)^*^	0.69 (0.46;1.04)
SNC	0.93 (0.68;1.28)	0.90 (0.65;1.26)	0.68 (0.46;1.02)	0.71 (0.47;1.08)
SNAP	0.98 (0.66;1.45)	0.94 (0.62;1.42)	0.63 (0.37;1.07)	0.59 (0.34;1.02)
SNCV	1.00 (0.72;1.40)	1.00 (0.71;1.40)	0.76 (0.51;1.13)	0.79 (0.52;1.19)
ESC – feet	1.11 (0.52;2.38)	1.16 (0.53;2.52)	1.02 (0.41;2.55)	0.99 (0.36;2.71)
**Continuous outcomes**	**Estimate (95% CI)**			
VPT	−0.15 (−5.44;5.43)	−0.16 (−5.47;5.44)	−0.78 (−6.62;5.43)	−0.63 (−6.46;5.55)
SNAP	0.38 (−6.40;7.65)	1.48 (−4.70;8.07)	5.62 (−0.78;12.42)	6.75 (0.35;13.56)^*^
SNCV	0.12 (−0.55;0.79)	0.06 (−0.59;0.72)	0.65 (0.01;1.30)^*^	0.64 (0.0;1.29)
ESC—hands	0.51 (−1.52;2.58)	−0.01 (−0.02;0.01)	0.29 (−1.93;2.55)	0.48 (−1.75;2.77)
ESC—feet	0.26 (−0.88;1.42)	0.49 (−1.52;2.55)	0.27 (−0.98;1.53)	0.47 (−0.78;1.73)

*Results are presented as odds ratios for binary outcomes based on logistic regression analyses and estimates for continuous outcomes based on linear regression analyses. Odds ratios show the change in odds for any increase of the GV determinants. Estimates show the percentage change in the outcomes for every 1-unit change of CV*.

*Model 1: unadjusted. Model 2: adjusted for age and gender. Model 3: adjusted for age, gender, HbA_1c_, diabetes duration, BMI, and exercise. Model 4: adjusted for age, gender, HbA_1c_, diabetes duration, BMI, exercise, systolic blood pressure, triglycerides, LDL cholesterol, and current smoking. The continuous outcomes of SDNN, RMSSD, LF, HF, LF/HF ratio, Total, VPT, SNAP, and ESC for hands and feet are log-transformed prior to analysis and subsequently back transformed to original scale. SDNN, RMSSD, LF, HF, LF/HF ratio, and Total are adjusted for HR in every model. Outcomes of DSPN are defined as presence of symmetric abnormal results. Binary outcomes were only included in the analyses if presence of five or more abnormal events*.

*CAN, Cardiovascular autonomic neuropathy; RMSSD, root mean square of the sum of the squares of differences between consecutive R–R intervals; SDNN, standard deviation of normal-to-normal intervals; LF/HF ratio, low-frequency power/high-frequency power ratio; DSPN, distal symmetric polyneuropathy; VPT, vibration perception threshold; SNC, sural nerve conduction; SNAP, sural nerve amplitude potential; SNCV, sural nerve conduction velocity; ESC, Electrochemical skin conduction*.

* *P < 0.05*.

#### Continuous Overall Net Glycemic Action (CONGA)

In the unadjusted model 1 higher CONGA was significantly associated to increasing incidents of subclinical DSPN, symmetric abnormalities SNAP, SNCV, and the composite measure SNC. Moreover, higher CONGA was associated to decreasing levels of the continuous measure of SNCV. When adjusted for gender and age in model 2 significance was kept for every outcome and in addition higher CONGA was associated with an increased risk of definite CAN. However, for every estimate significance was lost when adjusted for diabetes duration, BMI, and exercise and HbA_1c_ in model 3 (Table A1).

#### Mean Amplitude of Glucose Excursions (MAGE)

Greater MAGE became significantly associated with higher continuous measures of HRV only in the fully adjusted model 4 (Table A2) but when correcting for multiple tests significance was lost.

#### Time Spent in Hypo-, Eu-, and Hyperglycemia

None of the determinants “time spent in hypo-, eu-, and hyperglycemia” were significantly associated with diabetic neuropathy (Tables A3–A5).

#### Association Between HbA_1c_ and Diabetic Neuropathy

**Figure 3:**
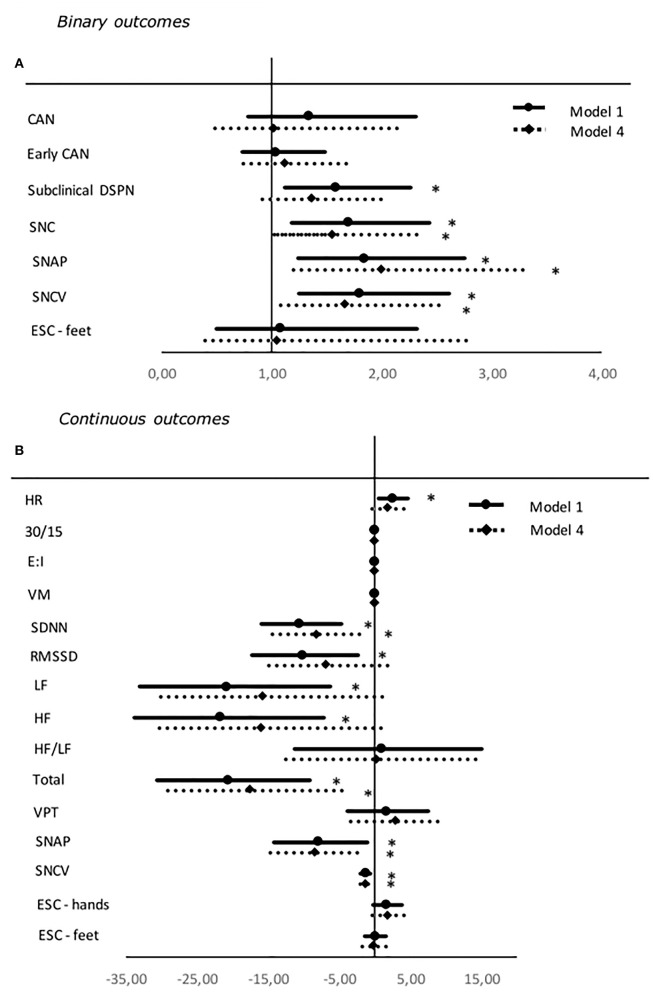
Forest plot of the associations between standardized values of HbA_1c_ and both binary **(A)** and continuous **(B)** neuropathy endpoints. For binary outcomes results are presented as odds ratio and 95% confidence intervals. Odds ratio shows the change in odds for an increase of one deviation in the HbA_1c_. For continuous outcomes results are presented as estimates and 95% confidence intervals. Estimates show the percentage change in the outcomes for an increase of one standard deviation in the SD. Studies with confidence interval crossing the vertical line are inconclusive. Model 1: unadjusted. Model 4: adjusted for age, gender, diabetes duration, BMI, exercise, systolic blood pressure, triglycerides, LDL cholesterol, and current smoking. SDNN, RMSSD, LF, HF, LF/HF ratio, and total are adjusted for HR in every model. Outcomes of DSPN are define as presence of symmetric abnormal results. Binary outcomes were only included in the analyses if presence of five or more abnormal events. CAN, Cardiovascular autonomic neuropathy; SNC, sural nerve conduction; SNAP, sural nerve amplitude potential; SNCV, sural nerve conduction velocity; ESC, Electrochemical skin conduction; RMSSD, root mean square of the sum of the squares of differences between consecutive R–R intervals; SDNN, standard deviation of normal-to-normal intervals; LF/HF ratio, low-frequency power/high-frequency power ratio; VPT, vibration perception threshold. *P < 0.05.

An increase in HbA_1c_ was associated with higher odds of subclinical DSPN when adjusted for diabetes duration, BMI, and exercise in model 3. Higher HbA_1c_ was significantly associated with increasing incidents of symmetric abnormalities in SNAP, SNCV, and the composite measure SNC in the fully adjusted model 4. Congruently, higher HbA_1c_ was associated to decreasing continuous values of SNAP and SNCV in model 4. Also, increasing levels of HbA_1c_ were associated with an increase in heart rate (HR) in model 3 and decreasing measures of HRV in model 4 (Table 5, Figure 3).

**Table 5:** The association between HbA_1c_ and measures of diabetic neuropathy.

**CAN measures**	**Model 1**	**Model 2**	**Model 3**	**Model 4**
**Binary outcomes**	**OR (95% CI)**			
CAN	1.02 (0.99;1.05)	1.02 (0.99;1.05)	1.01 (0.99;1.04)	1.01 (0.97;1.05)
Early CAN	1.0 (0.98;1.02)	1.0 (0.98;1.02)	1.0 (0.98;1.03)	1.01 (0.98;1.03)
**Continuous outcomes**	**Estimate (95% CI)**			
Heart rate	0.14 (0.03;0.26)^*^	0.14 (0.03;0.26)^*^	0.13 (0.02;0.25)^*^	0.11 (−0.02;0.24)
Lying to standing (30:15)	0.0 (0.0;0.0)	0.0 (0.0;0.0)	0.0 (0.0;0.0)	0.0 (0.0;0.0)
Deep breathing (E:I)	0.0 (0.0;0.0)	0.0 (0.0;0.0)	0.0 (0.0;0.0)	0.0 (0.0;0.0)
Valsalva Maneuver (VM)	0.0 (0.0;0.0)	0.0 (0.0;0.0)	0.0 (0.0;0.0)	0.0 (0.0;0.01)
SDNN	−0.68 (−1.02;−0.34)^*^	−0.67 (−1.01;−0.32)^*^	−0.65 (−0.99;−0.30)^*^	−0.55 (−0.94;−0.17)^*^
RMSSD	−0.66 (−1.11;−0.20)^*^	−0.65 (−1.11;−0.20)^*^	−0.63 (−1.09;−0.18)^*^	−0.46 (−0.97;0.04)
LF	−1.48 (−2.39;−0.56)^*^	−1.44 (−2.36;−0.52)^*^	−1.38 (−2.29;−0.46)^*^	−1.19 (−2.21;−0.17)^*^
HF	−1.48 (−2.39;−0.55)^*^	−1.46 (−2.37;−0.54)^*^	−1.41 (−2.33;−0.49)^*^	−1.11 (−2.13;−0.08)^*^
LF/HF ratio	0 (−0.72;0.72)	0.02 (−0.69;0.73)	0.03 (−0.65;0.72)	−0.08 (−0.83;0.67)
Total	−1.41 (−2.14;−0.68)^*^	−1.40 (−2.14;−0.66)^*^	−1.37 (−2.11;−0.63)^*^	−1.23 (−2.05;−0.40)^*^
**DSPN Measures**				
**Binary outcomes**	**OR (95% CI)**			
Subclinical DSPN	1.03 (1.01;1.05)^*^	1.03 (1.01;1.05)^*^	1.03 (1.01;1.05)^*^	1.02 (1.0;1.05)
SNC	1.03 (1.01;1.06)^*^	1.04 (1.01;1.06)^*^	1.03 (1.01;1.06)^*^	1.03 (1.01;1.06)^*^
SNAP	1.04 (1.01;1.06)^*^	1.04 (1.01;1.06)^*^	1.04 (1.02;1.07)^*^	1.04 (1.02;1.07)^*^
SNCV	1.04 (1.02;1.06)^*^	1.04 (1.02;1.06)^*^	1.04 (1.01;1.06)^*^	1.03 (1.01;1.06)^*^
ESC—feet	1.01 (0.97;1.05)	1.01 (0.97;1.05)	1.01 (0.96;1.05)	1.01 (0.96;1.06)
**Continuous outcomes**	**Estimate (95% CI)**			
VPT	0.09 (−0.22;0.40)	0.09 (−0.23;0.40)	0.10 (−0.22;0.42)	0.16 (−0.19;0.51)
SNAP	−0.54 (−0.93;−0.14)^*^	−0.46 (−0.83;−0.10)^*^	−0.46 (−0.81;−0.11)^*^	−0.61 (−1.0;−0.22)^*^
SNCV	−0.09 (−0.12;−0.05)^*^	−0.09 (−0.13;−0.06)^*^	−0.09 (−0.13;−0.05)^*^	−0.08 (−0.13;−0.04)^*^
ESC—hands	0.10 (−0.02;0.21)	0.10 (−0.01;0.21)	0.11 (−0.01;0.22)	0.10 (−0.02;0.23)
ESC—feet	−0.02 (−0.10;0.07)	−0.02 (−0.10;0.07)	−0.01 (−0.10;0.07)	−0.03 (−0.13;0.07)

*Results are presented as odds ratios for binary outcomes based on logistic regression analyses and estimates for continuous outcomes based on linear regression analyses. Odds ratios show the change in odds for any increase of the GV determinants. Estimates show the percentage change in the outcomes for every 1-unit change of CV*.

*Model 1: unadjusted. Model 2: adjusted for age and gender. Model 3: adjusted for age, gender, HbA_1c_, diabetes duration, BMI, and exercise. Model 4: adjusted for age, gender, HbA_1c_, diabetes duration, BMI, exercise, systolic blood pressure, triglycerides, LDL cholesterol, and current smoking. The continuous outcomes of SDNN, RMSSD, LF, HF, LF/HF ratio, Total, VPT, SNAP, and ESC for hands and feet are log-transformed prior to analysis and subsequently back transformed to original scale. SDNN, RMSSD, LF, HF, LF/HF ratio, and Total are adjusted for HR in every model. Outcomes of DSPN are defined as presence of symmetric abnormal results. Binary outcomes were only included in the analyses if presence of five or more abnormal events*.

*CAN, Cardiovascular autonomic neuropathy; RMSSD, root mean square of the sum of the squares of differences between consecutive R–R intervals; SDNN, standard deviation of normal-to-normal intervals; LF/HF ratio, low-frequency power/high-frequency power ratio; DSPN, distal symmetric polyneuropathy; VPT, vibration perception threshold; SNC, sural nerve conduction; SNAP, sural nerve amplitude potential; SNCV, sural nerve conduction velocity; ESC, Electrochemical skin conduction*.

* *P < 0.05*.

## Discussion

In this cross-sectional study 133 young adults with type 1 diabetes were identified with modest GV (38) with a mean coefficient of variation (CV) of 40% (38).

Modest associations between GV and measures of peripheral and autonomic diabetic neuropathy were found in the study. Higher CV was, against expectations, associated with decreased risk of DSPN and CAN, although not statistically significant when adjusting for relevant risk factors and multiple tests. Higher CONGA was associated with increasing incidents of both peripheral and autonomic neuropathy, but findings were confounded by relevant risk factors for diabetic neuropathy. After adjusting for risk factors higher MAGE was significantly associated with a slight increase in measures of HRV indicating an improvement of CAN. This may just be spurious findings—notably when the significant findings were attenuated after correcting for multiple tests. Overall only modest associations were found between GV and DSPN and CAN. Associations were confounded by known risk factors.

However, significant associations were found between higher levels of HbA_1c_ and increased risks of both peripheral and autonomic neuropathy in fully adjusted models. This only supports earlier findings of high levels of HbA_1c_ being an established and essential risk factor of diabetic neuropathy in type 1 diabetes (41).

Previous studies have revealed conflicting conclusions when examining the association between GV and diabetic neuropathy. Lachin et al. (17) evaluated 1,441 participants with type 1 diabetes and a mean age of 27 years from the DCCT. CARTs were performed every 2 years to evaluate CAN. GV was assessed by SD, MAGE, and *M*-value (a hybrid of glucose exposure and glycemic variability) (42). No significant associations were found between GV and CAN after correcting for within-day and longitudinal mean blood glucose and multiple tests. However, in the DCCT the GV parameters were calculated from 7 fingerstick glucose levels per day and this may have been an inadequate metrics of GV to detect GV's association with CAN.

Jaiswal et al. (16) found modest associations between GV and CAN. The study included 44 participants with type 1 diabetes and a mean age of 34 years. CAN was assessed by CARTs and HRV. Five days CGM was used to compute GV measures: low blood glucose index (LBGI) and area under the curve (AUC) for hypoglycemia. Significant inverse associations were found between both LBGI and AUC and the HF and LF power which implicates impaired autonomic function when longer and more severe hypoglycemia. However, when adjusting for relevant risk factors significance was attenuated. No relationship was identified between GV and any of the CARTs. Moreover, Kwai et al. (14) found significant associations between MAGE, assessed from 6-days CGM, and median motor and sensory excitability assessment in a study comprising 17 participants with type 1 diabetes and a mean age of 28.6 years. The findings of the study point at impairment of peripheral neuropathy induced by higher GV.

Nyiraty et al. (18) investigated the association between GV and autonomic neuropathy (AN) in 20 participants with type 1 diabetes and a mean age of 39.5 years. Fifty percent of the participants were diagnosed with CAN. AN was evaluated by CARTs and orthostatic blood pressure assessment. GV was assessed by 6 days of CGM and calculation of SD, MAGE, CONGA and mean absolute glucose (MAG). Only a correlation between SD and orthostatic hypotension was significant after adjusting for relevant risk factors. Again, the study does not provide a clear conclusion on the relationship between diabetic neuropathy and GV.

Studies investigating the association between GV and diabetic neuropathy in type 2 diabetes do not present uniform results. The number of studies investigating the association is limited and in particular studies assessing GV by CGM. However, CV in CGM was previously found to increase the risk of CAN in type 2 diabetes (23). Moreover, MAGE was significantly associated to DPN (24). Other studies have identified significant relations between variability in HbA1c and both DSPN and CAN (21–23). This may indicate an association between GV and diabetic neuropathy in type 2 diabetes however, like for studies concerning type 1 diabetes, there is a need for more studies with comparable measures of GV.

The results of our study are pointing at GV not being a risk factor for developing CAN and DSPN. However, the study has its limitations making causal conclusions difficult. We did find a significant association between higher levels of HbA_1c_ and CAN and DSPN which to some extent shows that poor glycemic control is indeed a risk factor for diabetic neuropathy.

## Strengths and Limitations

The cross-sectional design of the study is not ideal when examining the relationship between GV and diabetic neuropathy in a causal manner. A prospective observational study design would have been more appropriate.

As the study comprised 133 young adults identified with modest GV and prevalence of diabetic neuropathy there may not be enough power to extrapolate the results to the overall young population with type 1 diabetes. A larger sample size would have been beneficial in order to draw a more valid conclusion.

The aim of the study was to investigate the association between GV and early possible reversible neuropathy in a young cohort. Thus, conclusions on associations are limited to type 1 diabetes patients in the age-rage of the study cohort.

Novel and established methods of detecting DSPN and CAN (2, 35) were used which is a considerable strength in our study and may give a more detailed description of the nerve function.

CGM was used to assess GV as recommended (38) in order to detect periods of acute hypo- and hyperglycemia. However, the participants were only asked to wear the sensors for 5 days which may not be a sufficient duration to present a representable picture of daily fluctuations of blood glucose. Longer measuring durations may have given a more nuanced understanding.

It may be possible that more resourceful young adults chose to participate in the study after receiving the written invitation which could have caused selections bias.

It is recommended that participants avoid test confounders as smoking, use of several drugs, meals, and caffeine-containing liquids before testing for CAN (26). The participants were advised not to drink caffeine-containing liquids on the day before testing but the other recommendations were not met and may have affected CAN measures (4).

## Conclusion

After adjusting for relevant risk factors and multiple tests, no significant associations were found between GV and diabetic neuropathy in a cohort of young adults with type 1 diabetes. This finding is in line with some of the previous studies which have failed to provide consistent evidence that GV is a risk factor of development of CAN and DSPN.

This suggests that GV may not be a risk factor for early diabetic neuropathy in young adults with type 1 diabetes. However, the cross-sectional study approach including a relatively small sample size of young participants with modest GV and diabetic neuropathy make a strong conclusion difficult. Moreover, long-term effects of GV excursions may still play a role in the pathogenic mechanisms leading to neuropathy in later life. Increasing levels of HbA_1c_ were significantly associated with both measures of DSPN and CAN which support earlier findings of high levels of HbA_1c_ being an established and essential risk factor of diabetic neuropathy.

Previous studies addressing the aim of the present project have assessed GV and DSPN and CAN by heterogenic measuring modalities hampering comparability. To improve comparability there is a need for studies using recommended measures of GV and diabetic neuropathy. Furthermore, more studies on young adults with type 1 diabetes are needed to confirm our findings.

## Data Availability Statement

The raw data supporting the conclusions of this article will be made available by the authors, without undue reservation.

## Ethics Statement

The studies involving human participants were reviewed and approved by The Danish Research Ethics Committee (project id.: H-15006967). The patients/participants provided their written informed consent to participate in this study.

## Author Contributions

MC has contributed to the design of the study, acquired, analyzed, interpreted data, drafted the article, and approved the final version to be published. EH, MJ, and JF has contributed to the design of the study, analyzed, interpreted data, revised the article critically, and approved the final version to be published. CH has contributed to the design of the study, acquisition, analysis, interpretation of data, revised the article critically, and approved the final version to be published. All authors contributed to the article and approved the submitted version.

## Conflict of Interest

JF holds stocks in Medicus Engineering. The remaining authors declare that the research was conducted in the absence of any commercial or financial relationships that could be construed as a potential conflict of interest.

## Abbreviations

BPI: Brief Pain Inventory
CAN: cardiovascular autonomic neuropathy
CARTs: cardiovascular autonomic reflex tests
CONGA: continuous overall net glycemic action
CSII: continuous subcutaneous insulin infusion
CV: coefficient of variation
DSPN: diabetic symmetric polyneuropathy
E:I: deep breathing test
ESC: electrochemical skin conductance
HF: high frequency
HRV: heart rate variability
IQR: interquartile range
LF: low frequency
LF/HF: ratio low-frequency power/high-frequency power ratio
MAGE: mean amplitude of glucose excursions
MDI: multiple dose injections
MNSI: Michigan Neuropathy Screening Instrument
RMSSD: root mean square of the sum of the squares of differences between consecutive R–R intervals
SDNN: standard deviation of normal-to-normal intervals
SNAP: sural nerve amplitude potential
SNCV: sural nerve conduction velocity
VM: Valsalva Maneuver
VPT: vibration perception threshold
30:15: lying-to-standing test.
