# Supplementary material for: Glycemic Variability and Diabetic Neuropathy in Young Adults With Type 1 Diabetes
Source: Front Endocrinol (Lausanne). 2020 Sep 23;11:644. doi: 10.3389/fendo.2020.00644 (PMC7538646; doi:10.3389/fendo.2020.00644)
Supplement: Supplementary file 1 [file Table_1.DOCX]

| **CAN measures** | **Model 1** | **Model 2** | **Model 3** | **Model 4** |
| --- | --- | --- | --- | --- |
| ***Binary outcomes*** | ***OR (95% CI)*** | | | |
| CAN | 1.32 (1.0;1.75) | 1.39 (1.02;1.88)* | 1.11 (0.67;1.84) | 1.20 (0.71;2.02) |
| Early CAN | 0.98 (1.0;1.75) | 0.98 (0.82;1.18) | 1.09 (0.82;1.44) | 1.13 (0.85;1.51) |
| ***Continuous outcomes*** | ***Estimate (95% CI)*** | | | |
| Heart rate | 0.71 (-0.31;1.73) | 0.71 (-0.30;1.72) | -0.33 (-1.79;1.14) | -0.29 (-1.75;1.17) |
| Lying to standing (30:15) | -0.01 (-0.03;0.0) | -0.01 (-0.03;0.0) | 0.0 (-0.03;0.02) | 0.0 (-0.03;0.02) |
| Deep breathing (E:I) | 0.0 (-0.02;0.02) | 0.0 (-0.02;0.02) | 0.02 (-0.01;0.05) | 0.01 (-0.01;0.04) |
| Valsalva Manoeuvre (VM) | 0.02 (-0.01;0.05) | 0.02 (-0.01;0.04) | 0.01 (-0.03;0.05) | 0.01 (-0.03;0.05) |
| SDNN | -2.04 (-4.95;0.96) | -1.94 (-4.87;1.09) | 2.52 (-1.81;7.03) | 3.25 (-1.13;7.83) |
| RMSSD | -1.72 (-5.47;2.19) | -1.72 (-5.48;2.18) | 3.36 (-2.26;9.30) | 3.99 (-1.72;10.02) |
| LF | -2.65 (-10.19;5.52) | -2.36 (-9.96;5.88) | 8.02 (-3.61;21.05) | 10.62 (-1.33;24.01) |
| HF | -2.89 (-10.39;5.24) | -2.82 (-10.29;5.28) | 8.52 (-3.17;21.62) | 9.51 (-2.36;22.83) |
| LF/HF ratio | 0.25 (-5.83;6.71) | 0.47 (-5.48;6.81) | -0.46 (-8.58;8.38) | 1.01 (-7.21;9.96) |
| Total | -3.83 (-2.92;2.66) | -3.71 (-9.84;2.84) | 6.58 (-2.91;16.99) | 8.45 (-1.28;19.14) |
| **DSPN measures** |  |  |  |  |
| ***Binary outcomes*** | ***OR (95% CI)*** | | | |
| Subclinical DSPN | 1.21 (1.02;1.44)* | 1.21 (1.02;1.44)* | 1.13 (0.88;1.44) | 1.13 (0.88;1.47) |
| SNC | 1.23 (1.03;1.46)* | 1.23 (1.03;1.48)* | 1.13 (0.88;1.46) | 1.14 (0.87;1.48) |
| SNAP | 1.31 (1.06;1.60)* | 1.29 (1.03;1.60)* | 1.02 (0.74;1.39) | 0.98 (0.70;1.35) |
| SNCV | 1.24 (1.04,1.49)* | 1.25 (1.04;1.50)* | 1.11 (0.86;1.44) | 1.12 (0.85;1.46) |
| ESC - feet | 1.0 (0.69;1.45) | 1.03 (0.69;1.54) | 1.11 (0.56;2.17) | 1.06 (0.50;2.27) |
| ***Continuous outcomes*** | ***Estimate (95% CI)*** | | | |
| VPT | 2.55 (-0.11;5.27) | 2.59 (-0.09;5.33) | 3.72 (-0.27;7.87) | 3.47 (-0.49;7.59) |
| SNAP | -3.04 (-6.26;0.29) | -2.40 (-5.34;0.64) | 1.23 (-2.89;5.52) | 1.93 (-2.19;6.24) |
| SNCV | -0.37 (-0.69;-0.05)* | -0.41 (-0.72;-0.10)* | -0.10 (-0.53;0.33) | -0.05 (-0.48;0.38) |
| ESC - hands | 0.15 (-0.85;1.15) | 0.15 (-0.84;1.14) | -0.43 (-1.87;1.04) | -0.24 (-1.70;1.24) |
| ESC - feet | -0.05 (-0.62;0.51) | -0.07 (-0.64;0.50) | -0.52 (-1.34;0.31) | -0.40 (-1.22;0.43) |

# **Appendix**

**Table A1** The association between CONGA and measures of diabetic neuropathy.

Results are presented as odds ratios for binary outcomes based on logistic regression analyses and estimates for continuous outcomes based on linear regression analyses. Odds ratios show the change in odds for any increase of the GV determinants. Estimates show the percentage change in the outcomes for every 1-unit change of CV.

Model 1: unadjusted. Model 2: adjusted for age and gender. Model 3: adjusted for age, gender, HbA_1c_, diabetes duration, BMI and exercise. Model 4: adjusted for age, gender, HbA_1c_, diabetes duration, BMI, exercise, systolic blood pressure, triglycerides, LDL cholesterol and current smoking. The continuous outcomes of SDNN, RMSSD, LF, HF, LF/HF ratio, Total, VPT, SNAP and ESC for hands and feet are log-transformed prior to analysis and subsequently back transformed to original scale. SDNN, RMSSD, LF, HF, LF/HF ratio and Total are adjusted for HR in every model. Outcomes of DSPN are defined as presence of symmetric abnormal results. Binary outcomes were only included in the analyses if presence of 5 or more abnormal events.

*CAN:* Cardiovascular autonomic neuropathy, *RMSSD:* root mean square of the sum of the squares of differences between consecutive R–R intervals, *SDNN:* standard deviation of normal-to-normal intervals, *LF/HF ratio:* low-frequency power/high-frequency power ratio, *DSPN:* distal symmetric polyneuropathy*,* *VPT:* vibration perception threshold, *SNC:* sural nerve conduction*, SNAP:* sural nerve amplitude potential, *SNCV:* sural nerve conduction velocity, *ESC:* Electrochemical skin conduction.

* P < 0.05.

| **CAN measures** | **Model 1** | **Model 2** | **Model 3** | **Model 4** |
| --- | --- | --- | --- | --- |
| ***Binary outcomes*** | ***OR (95% CI)*** | | | |
| CAN | 1.0 (0.98;1.03) | 1.0 (0.97;1.03) | 1.0 (0.98;1.03) | 1.0 (0.97;1.03) |
| Early CAN | 0.99 (0.96;1.02) | 0.99 (0.96;1.02) | 0.99 (0.97;1.02) | 0.99 (0.97;1.02) |
| ***Continuous outcomes*** | ***Estimate (95% CI)*** | | | |
| Heart rate | 0.01 (0.0;0.03) | 0.0 (-0.01;0.0) | 0.01 (-0.01;0.02) | 0.0 (-0.01;0.02) |
| Lying to standing (30:15) | 0.0 (0.0;0.0) | 0.0 (0.0;0.0) | 0.0 (0.0;0.0) | 0.0 (0.0;0.0) |
| Deep breathing (E:I) | 0.0 (0.0;0.0) | 0.0 (0.0;0.0) | 0.0 (0.0;0.0) | 0.0 (0.0;0.0) |
| Valsalva Manoeuvre (VM) | 0.0 (0.0;0.0) | 0.0 (0.0;0.0) | 0.0 (0.0;0.0) | 0.0 (0.0;0.0) |
| SDNN | 0.02 (-0.03;0.06) | 0.02 (-0.02;0.07) | 0.06 (0.0;0.11) | 0.06 (0.01;0.11)* |
| RMSSD | 0.03 (-0.02;0.09) | 0.03 (-0.02;0.09) | 0.07 (0.0;0.14) | 0.08 (0.01;0.11)* |
| LF | 0.01 (-0.11;0.13) | 0.02 (-0.10;0.14) | 0.11 (-0.03;0.25) | 0.13 (-0.01;0.27) |
| HF | 0.12 (0.0;0.23) | 0.12 (0.0;0.24) | 0.18 (0.04;0.32)* | 0.19 (0.05;0.33)* |
| LF/HF ratio | -0.11 (-0.20;-0.02)* | -0.10 (-0.19;-0.02)* | -0.07 (-0.18;0.32) | -0.07 (-0.17;0.04) |
| Total | 0.04 (-0.06;0.13) | 0.04 (-0.06;0.14) | 0.12 (0.0;0.23) | 0.13 (0.02;0.24)* |
| **DSPN measures** |  |  |  |  |
| ***Binary outcomes*** | ***OR (95% CI)*** | | | |
| Subclinical DSPN | 1.01 (0.99;1.02) | 1.01 (0.99;1.02) | 1.0 (0.99;1.02) | 1.0 (0.99;1.02) |
| SNC | 1.0 (0.96;1.05) | 1.01 (0.99;1.02) | 1.01 (0.99;1.02) | 1.0 (0.99;1.02) |
| SNAP | 1.0 (0.98;1.03) | 1.0 (0.99;1.02) | 1.0 (0.99;1.02) | 1.0 (0.99;1.02) |
| SNCV | 1.01 (0.98;1.03) | 1.01 (0.98;1.03) | 1.0 (0.99;1.02) | 1.0 (0.99;1.02) |
| ESC - feet | 1.0 (0.98;1.03) | 1.0 (0.98;1.03) | 1.0 (0.97;1.03) | 1.0 (0.97;1.03) |
| ***Continuous outcomes*** | ***Estimate (95% CI)*** | | | |
| VPT | 0.01 (-0.03;0.05) | 0.01 (-0.03;0.05) | 0.01 (-0.04;0.06) | 0.01 (-0.04;0.06) |
| SNAP | 0.01 (-0.04;0.06) | 0.02 (-0.03;0.06) | 0.03 (-0.02;0.08) | 0.04 (-0.01;0.09) |
| SNCV | 0.0 (0.0;0.0) | 0.0 (-0.01;0.0) | 0.0 (-0.01;0.0) | 0.0 (-0.01;0.01) |
| ESC - hands | -0.01 (-0.02;0.01) | -0.01 (-0.02;0.01) | -0.01 (-0.03;0.0) | -0.01 (-0.03;0.0) |
| ESC - feet | 0.0 (-0.01;0.01) | 0.0 (-0.01;0.01) | 0.0 (-0.01;0.01) | 0.0 (-0.01;0.01) |

**Table A2** The association between MAGE and measures of diabetic neuropathy.

Results are presented as odds ratios for binary outcomes based on logistic regression analyses and estimates for continuous outcomes based on linear regression analyses. Odds ratios show the change in odds for any increase of the GV determinants. Estimates show the percentage change in the outcomes for every 1-unit change of CV.

Model 1: unadjusted. Model 2: adjusted for age and gender. Model 3: adjusted for age, gender, HbA_1c_, diabetes duration, BMI and exercise. Model 4: adjusted for age, gender, HbA_1c_, diabetes duration, BMI, exercise, systolic blood pressure, triglycerides, LDL cholesterol and current smoking. The continuous outcomes of SDNN, RMSSD, LF, HF, LF/HF ratio, Total, VPT, SNAP and ESC for hands and feet are log-transformed prior to analysis and subsequently back transformed to original scale. SDNN, RMSSD, LF, HF, LF/HF ratio and Total are adjusted for HR in every model. Outcomes of DSPN are defined as presence of symmetric abnormal results. Binary outcomes were only included in the analyses if presence of 5 or more abnormal events.

*CAN:* Cardiovascular autonomic neuropathy, *RMSSD:* root mean square of the sum of the squares of differences between consecutive R–R intervals, *SDNN:* standard deviation of normal-to-normal intervals, *LF/HF ratio:* low-frequency power/high-frequency power ratio, *DSPN:* distal symmetric polyneuropathy*,* *VPT:* vibration perception threshold, *SNC:* sural nerve conduction*, SNAP:* sural nerve amplitude potential, *SNCV:* sural nerve conduction velocity, *ESC:* Electrochemical skin conduction.

* P < 0.05.

| **CAN measures** | **Model 1** | **Model 2** | **Model 3** | **Model 4** |
| --- | --- | --- | --- | --- |
| ***Binary outcomes*** | ***OR (95% CI)*** | | | |
| CAN | 0.99 (0.98;1.0) | 0.99 (0.98;1.0) | 1.0 (0.99;1.0) | 0.99 (0.99;1.0) |
| Early CAN | 1.0 (1.0;1.0) | 1.0 (1.0;1.0) | 1.0 (1.0;1.0) | 1.0 (1.0;1.0) |
| ***Continuous outcomes*** | ***Estimate (95% CI)*** | | | |
| Heart rate | 0.0 (-0.01;0.01) | 0.0 (-0.01;0.01) | 0.0 (-0.01;0.01) | 0.0 (-0.01;0.01) |
| Lying to standing (30:15) | 0.0 (0.0;0.0) | 0.0 (0.0;0.0) | 0.0 (0.0;0.0) | 0.0 (0.0;0.0) |
| Deep breathing (E:I) | 0.0 (0.0;0.0) | 0.0 (0.0;0.0) | 0.0 (0.0;0.0) | 0.0 (0.0;0.0) |
| Valsalva Manoeuvre (VM) | 0.0 (0.0;0.0) | 0.0 (0.0;0.0) | 0.0 (0.0;0.0) | 0.0 (0.0;0.0) |
| SDNN | 0.0 (-0.04;0.03) | 0.0 (-0.04;0.03) | -0.01 (-0.05;0.02) | -0.01 (-0.05;0.02) |
| RMSSD | -0.01 (-0.06;0.03) | -0.01 (-0.05;0.03) | -0.02 (-0.06;0.02) | -0.02 (-0.07;0.02) |
| LF | -0.01 (-0.10;0.08) | -0.01 (-0.10;0.08) | -0.02 (-0.12;0.07) | -0.03 (-0.12;0.06) |
| HF | -0.04 (-0.13;0.04) | -0.04 (-0.13;0.04) | -0.06 (-0.15;0.03) | -0.06 (-0.15;0.03) |
| LF/HF ratio | 0.03 (-0.03;0.10) | 0.03 (-0.03;0.10) | 0.03 (-0.03;0.10) | 0.03 (-0.04;0.10) |
| Total | -0.01 (-0.08;0.06) | -0.01 (-0.08;0.07) | -0.02 (-0.10;0.05) | -0.03 (-0.10;0.05) |
| **DSPN measures** |  |  |  |  |
| ***Binary outcomes*** | ***OR (95% CI)*** | | | |
| Subclinical DSPN | 1.0 (1.0;1.0) | 1.0 (1.0;1.0) | 1.0 (1.0;1.0) | 1.0 (1.0;1.0) |
| SNC | 1.0 (1.0;1.0) | 1.0 (1.0;1.0) | 1.0 (1.0;1.0) | 1.0 (1.0;1.0) |
| SNAP | 1.0 (1.0;1.0) | 1.0 (1.0;1.0) | 1.0 (1.0;1.0) | 1.0 (1.0;1.0) |
| SNCV | 1.0 (1.0;1.0) | 1.0 (1.0;1.0) | 1.0 (1.0;1.0) | 1.0 (1.0;1.0) |
| ESC - feet | 1.0 (1.0;1.0) | 1.0 (1.0;1.01) | 1.0 (1.0;1.01) | 1.0 (1.0;1.01) |
| ***Continuous outcomes*** | ***Estimate (95% CI)*** | | | |
| VPT | -0.02 (-0.05;0.01) | -0.02 (-0.05;0.01) | -0.02 (-0.05;0.01) | -0.02 (-0.05;0.01) |
| SNAP | -0.01 (-0.04;0.03) | 0.0 (-0.04;0.03) | -0.02 (-0.05;0.01) | -0.02 (-0.05;0.01) |
| SNCV | 0.0 (0.0;0.01) | 0.0 (0.0;0.01) | 0.0 (0.0;0.01) | 0.0 (0.0;0.01) |
| ESC - hands | 0.0 (-0.01;0.01) | 0.0 (-0.01;0.01) | 0.0 (-0.01;0.01) | 0.0 (-0.01;0.01) |
| ESC - feet | 0.0 (-0.01;0.01) | 0.0 (-0.01;0.01) | 0.0 (-0.01;0.01) | 0.0 (-0.01;0.01) |

**Table A3** The association between time spent in hypoglycemia (<3.0 mmol/l) and measures of diabetic neuropathy.

Results are presented as odds ratios for binary outcomes based on logistic regression analyses and estimates for continuous outcomes based on linear regression analyses. Odds ratios show the change in odds for any increase of the GV determinants. Estimates show the percentage change in the outcomes for every 1-unit change of CV.

Model 1: unadjusted. Model 2: adjusted for age and gender. Model 3: adjusted for age, gender, HbA_1c_, diabetes duration, BMI and exercise. Model 4: adjusted for age, gender, HbA_1c_, diabetes duration, BMI, exercise, systolic blood pressure, triglycerides, LDL cholesterol and current smoking. The continuous outcomes of SDNN, RMSSD, LF, HF, LF/HF ratio, Total, VPT, SNAP and ESC for hands and feet are log-transformed prior to analysis and subsequently back transformed to original scale. SDNN, RMSSD, LF, HF, LF/HF ratio and Total are adjusted for HR in every model. Outcomes of DSPN are defined as presence of symmetric abnormal results. Binary outcomes were only included in the analyses if presence of 5 or more abnormal events.

*CAN:* Cardiovascular autonomic neuropathy, *RMSSD:* root mean square of the sum of the squares of differences between consecutive R–R intervals, *SDNN:* standard deviation of normal-to-normal intervals, *LF/HF ratio:* low-frequency power/high-frequency power ratio, *DSPN:* distal symmetric polyneuropathy*,* *VPT:* vibration perception threshold, *SNC:* sural nerve conduction*, SNAP:* sural nerve amplitude potential, *SNCV:* sural nerve conduction velocity, *ESC:* Electrochemical skin conduction.

* P < 0.05.

**Table A4** The association between time spent in euglycemia (≥3.0; ≤10.0 mmol/l) and measures of diabetic neuropathy.

| **CAN measures** | **Model 1** | **Model 2** | **Model 3** | **Model 4** |
| --- | --- | --- | --- | --- |
| ***Binary outcomes*** | ***OR (95% CI)*** | | | |
| CAN | 1.0 (1.0;1.0) | 1.0 (1.0;1.0) | 1.0 (1.0;1.0) | 1.0 (1.0;1.0) |
| Early CAN | 1.0 (1.0;1.0) | 1.0 (1.0;1.0) | 1.0 (1.0;1.0) | 1.0 (1.0;1.0) |
| ***Continuous outcomes*** | ***Estimate (95% CI)*** | | | |
| Heart rate | 0.0 (0.0;0.0) | 0.0 (0.0;0.0) | 0.0 (0.0;0.0) | 0.0 (0.0;0.0) |
| Lying to standing (30:15) | 0.0 (0.0;0.0) | 0.0 (0.0;0.0) | 0.0 (0.0;0.0) | 0.0 (0.0;0.0) |
| Deep breathing (E:I) | 0.0 (0.0;0.0) | 0.0 (0.0;0.0) | 0.0 (0.0;0.0) | 0.0 (0.0;0.0) |
| Valsalva Manoeuvre (VM) | 0.0 (0.0;0.0) | 0.0 (0.0;0.0) | 0.0 (0.0;0.0) | 0.0 (0.0;0.0) |
| SDNN | 0.01 (0.0;0.01) | 0.01 (0.0;0.01) | 0.0 (0;0.01) | 0.0 (0.0;0.01) |
| RMSSD | 0.01 (0.0;0.01) | 0.01 (0.0;0.01) | 0.0 (0;0.01) | 0.0 (0.0;0.01) |
| LF | 0.01 (0.0;0.03) | 0.01 (0.0;0.03) | 0.0 (-0.01;0.02) | 0 (-0.01;0.02) |
| HF | 0.02 (0.0;0.03) | 0.02 (0.0;0.03) | 0.01 (-0.01;0.03) | 0.01 (-0.01;0.03) |
| LF/HF ratio | 0.0 (-0.01;0.01) | 0.0 (-0.01;0.01) | -0.01 (-0.02;0.01) | -0.01 (-0.02;0) |
| Total | 0.01 (0.0;0.02) | 0.01 (0.0;0.02) | 0.01 (-0.01;0.02) | 0.01 (-0.01;0.02) |
| **DSPN measures** |  |  |  |  |
| ***Binary outcomes*** | ***OR (95% CI)*** | | | |
| Subclinical DSPN | 1.0 (1.0;1.0) | 1.0 (1.0;1.0) | 1.0 (1.0;1.0) | 1.0 (1.0;1.0) |
| SNC | 1.0 (1.0;1.0) | 1.0 (1.0;1.0) | 1.0 (1.0;1.0) | 1.0 (1.0;1.0) |
| SNAP | 1.0 (1.0;1.0) | 1.0 (1.0;1.0) | 1.0 (1.0;1.0) | 1.0 (1.0;1.0) |
| SNCV | 1.0 (1.0;1.0) | 1.0 (1.0;1.0) | 1.0 (1.0;1.0) | 1.0 (1.0;1.0) |
| ESC - feet | 1.0 (1.0;1.0) | 1.0 (1.0;1.0) | 1.0 (1.0;1.0) | 1.0 (1.0;1.0) |
| ***Continuous outcomes*** | ***Estimate (95% CI)*** | | | |
| VPT | 0.0 (-0.01;0.0) | 0.0 (-0.01;0.0) | 0.0 (-0.01;0.0) | 0.0 (-0.01;0.0) |
| SNAP | 0.01 (0.0;0.01) | 0.01 (0;0.01) | 0.0 (0.0;0.01) | 0.0 (0;0.01) |
| SNCV | 0.0 (0.0;0.0) | 0.0 (0.0;0.0) | 0.0 (0.0;0.0) | 0.0 (0.0;0.0) |
| ESC - hands | 0.0 (0.0;0.0) | 0.0 (0.0;0.0) | 0.0 (0.0;0.0) | 0.0 (0.0;0.0) |
| ESC - feet | 0.0 (0.0;0.0) | 0.0 (0.0;0.0) | 0.0 (0.0;0.0) | 0.0 (0.0;0.0) |

Results are presented as odds ratios for binary outcomes based on logistic regression analyses and estimates for continuous outcomes based on linear regression analyses. Odds ratios show the change in odds for any increase of the GV determinants. Estimates show the percentage change in the outcomes for every 1-unit change of CV.

Model 1: unadjusted. Model 2: adjusted for age and gender. Model 3: adjusted for age, gender, HbA_1c_, diabetes duration, BMI and exercise. Model 4: adjusted for age, gender, HbA_1c_, diabetes duration, BMI, exercise, systolic blood pressure, triglycerides, LDL cholesterol and current smoking. The continuous outcomes of SDNN, RMSSD, LF, HF, LF/HF ratio, Total, VPT, SNAP and ESC for hands and feet are log-transformed prior to analysis and subsequently back transformed to original scale. SDNN, RMSSD, LF, HF, LF/HF ratio and Total are adjusted for HR in every model. Outcomes of DSPN are defined as presence of symmetric abnormal results. Binary outcomes were only included in the analyses if presence of 5 or more abnormal events.

*CAN:* Cardiovascular autonomic neuropathy, *RMSSD:* root mean square of the sum of the squares of differences between consecutive R–R intervals, *SDNN:* standard deviation of normal-to-normal intervals, *LF/HF ratio:* low-frequency power/high-frequency power ratio, *DSPN:* distal symmetric polyneuropathy*,* *VPT:* vibration perception threshold, *SNC:* sural nerve conduction*, SNAP:* sural nerve amplitude potential, *SNCV:* sural nerve conduction velocity, *ESC:* Electrochemical skin conduction.

* P < 0.05.

| **CAN measures** | **Model 1** | **Model 2** | **Model 3** | **Model 4** |
| --- | --- | --- | --- | --- |
| ***Binary outcomes*** | ***OR (95% CI)*** | | | |
| CAN | 1.0 (1.0;1.0) | 1.0 (1.0;1.0) | 1.0 (1.0;1.0) | 1.0 (1.0;1.0) |
| Early CAN | 1.0 (1.0;1.0) | 1.0 (1.0;1.0) | 1.0 (1.0;1.0) | 1.0 (1.0;1.0) |
| ***Continuous outcomes*** | ***Estimate (95% CI)*** | | | |
| Heart rate | 0.0 (0.0;0.0) | 0.0 (0.0;0.0) | 0.0 (0.0;0.0) | 0.0 (0.0;0.0) |
| Lying to standing (30:15) | 0.0 (0.0;0.0) | 0.0 (0.0;0.0) | 0.0 (0.0;0.0) | 0.0 (0.0;0.0) |
| Deep breathing (E:I) | 0.0 (0.0;0.0) | 0.0 (0.0;0.0) | 0.0 (0.0;0.0) | 0.0 (0.0;0.0) |
| Valsalva Manoeuvre (VM) | 0.0 (0.0;0.0) | 0.0 (0.0;0.0) | 0.0 (0.0;0.0) | 0.0 (0.0;0.0) |
| SDNN | 0.0 (-0.01;0.0) | 0.0 (-0.01;0.0) | 0.0 (0.0;0.01) | 0.0 (0.0;0.01) |
| RMSSD | 0.0 (-0.01;0.0) | 0.0 (-0.01;0.0) | 0.0 (0.0;0.01) | 0.01 (0.0;0.01) |
| LF | 0.0 (-0.02;0.01) | 0.0 (-0.02;0.01) | 0.01 (-0.01;0.03) | 0.01 (0.0;0.03) |
| HF | -0.01 (-0.02;0.01) | -0.01 (-0.02;0.01) | 0.01 (-0.01;0.02) | 0.01 (-0.01;0.03) |
| LF/HF ratio | 0.0 (-0.01;0.01) | 0.0 (-0.01;0.01) | 0.0 (-0.01;0.02) | 0.0 (-0.01;0.02) |
| Total | 0.0 (-0.02;0.01) | 0.0 (-0.02;0.01) | 0.01 (-0.01;0.02) | 0.01 (0;0.03) |
| **DSPN measures** |  |  |  |  |
| ***Binary outcomes*** | ***OR (95% CI)*** | | | |
| Subclinical DSPN | 1.0 (1.0;1.0) | 1.0 (1.0;1.0) | 1.0 (1.0;1.0) | 1.0 (1.0;1.0) |
| SNC | 1.0 (1.0;1.0) | 1.0 (1.0;1.0) | 1.0 (1.0;1.0) | 1.0 (1.0;1.0) |
| SNAP | 1.0 (1.0;1.0) | 1.0 (1.0;1.0) | 1.0 (1.0;1.0) | 1.0 (1.0;1.0) |
| SNCV | 1.0 (1.0;1.0) | 1.0 (1.0;1.0) | 1.0 (1.0;1.0) | 1.0 (1.0;1.0) |
| ESC - feet | 1.0 (1.0;1.0) | 1.0 (1.0;1.0) | 1.0 (1.0;1.0) | 1.0 (1.0;1.0) |
| ***Continuous outcomes*** | ***Estimate (95% CI)*** | | | |
| VPT | 0.0 (0.0;0.01) | 0.0 (0.0;0.01) | 0.0 (0.0;0.01) | 0.0 (0.0;0.01) |
| SNAP | 0.0 (-0.01;0.0) | 0.0 (-0.01;0.0) | 0.0 (0.0;0.01) | 0.0 (0.0;0.01) |
| SNCV | 0.0 (0.0;0.0) | 0.0 (0.0;0.0) | 0.0 (0.0;0.0) | 0.0 (0.0;0.0) |
| ESC - hands | 0.0 (0.0;0.0) | 0.0 (0.0;0.0) | 0.0 (0.0;0.0) | 0.0 (0.0;0.0) |
| ESC - feet | 0.0 (0.0;0.0) | 0.0 (0.0;0.0) | 0.0 (0.0;0.0) | 0.0 (0.0;0.0) |

**Table A5** The association between time spent in hyperglycemia (>10.0 mmol/l) and measures of diabetic neuropathy.

Results are presented as odds ratios for binary outcomes based on logistic regression analyses and estimates for continuous outcomes based on linear regression analyses. Odds ratios show the change in odds for any increase of the GV determinants. Estimates show the percentage change in the outcomes for every 1-unit change of CV.

Model 1: unadjusted. Model 2: adjusted for age and gender. Model 3: adjusted for age, gender, HbA_1c_, diabetes duration, BMI and exercise. Model 4: adjusted for age, gender, HbA_1c_, diabetes duration, BMI, exercise, systolic blood pressure, triglycerides, LDL cholesterol and current smoking. The continuous outcomes of SDNN, RMSSD, LF, HF, LF/HF ratio, Total, VPT, SNAP and ESC for hands and feet are log-transformed prior to analysis and subsequently back transformed to original scale. SDNN, RMSSD, LF, HF, LF/HF ratio and Total are adjusted for HR in every model. Outcomes of DSPN are defined as presence of symmetric abnormal results. Binary outcomes were only included in the analyses if presence of 5 or more abnormal events.

*CAN:* Cardiovascular autonomic neuropathy, *RMSSD:* root mean square of the sum of the squares of differences between consecutive R–R intervals, *SDNN:* standard deviation of normal-to-normal intervals, *LF/HF ratio:* low-frequency power/high-frequency power ratio, *DSPN:* distal symmetric polyneuropathy*,* *VPT:* vibration perception threshold, *SNC:* sural nerve conduction*, SNAP:* sural nerve amplitude potential, *SNCV:* sural nerve conduction velocity, *ESC:* Electrochemical skin conduction.

* P < 0.05.

**Figure 2** Forest plot of the associations between standardized values of SD and both binary and continuous neuropathy endpoints
